# Botulinum Toxin Therapy in Writer’s Cramp and Musician’s Dystonia

**DOI:** 10.3390/toxins13120899

**Published:** 2021-12-14

**Authors:** Elina Zakin, David M. Simpson

**Affiliations:** 1 Department of Neurology, New York University Grossman School of Medicine, New York, NY 10017, USA; 2 Department of Neurology, Icahn School of Medicine at Mount Sinai, New York, NY 10029, USA; David.simpson@mssm.edu

**Keywords:** writer’s cramp, task-specific dystonia, botulinum toxin, musician’s dystonia

## Abstract

Task-specific focal dystonia is characterized by muscle contraction(s) during a specific task, resulting in abnormal postures or movements. Specifically, writer’s cramp involves the upper extremity during the act of writing. Musician’s dystonia has a highly variable presentation, and thus makes therapeutic options more limited. Treatments include oral pharmacologic agents, neuromodulation, surgery and, most often, botulinum toxin (BoNT) injection. Selection of target muscles for toxin injection continues to be an area of active research for these task-specific movements. We present a review of the literature selected from a predefined search of the *MEDLINE* and ClinicalTrials.gov databases. We include six controlled studies of botulinum toxin for the management of writer’s cramp and focal task-specific dystonia (FTSD), including musician’s dystonia. Overall, 139 patients were included across all studies, with 99 individuals injected for writer’s cramp and the remaining 40 individuals with FTSD. The age range of all patients was 18–80 years old. We included studies that utilized only the BoNT-A serotype. These studies utilized various severity scales to quantify response to toxin injection, with ratings of instrument or pen control included as subjective ratings. Of the included 139 patients in this review, pooled data for toxin response show that 73% of patients who received the drug demonstrated improvement. Specific techniques for muscle localization and targeting were difficult to study as variable methods were employed. This remains an area of ongoing exploration.

## 1. Introduction

Dystonia is characterized by sustained or intermittent muscle contractions causing abnormal, typically repetitive movements, postures or both [1]. Dystonia is classified into focal, multifocal, generalized, segmental or hemi-dystonia [2]. This review will focus on the utility of botulinum toxin (BoNT) in the management of task-specific focal dystonia, (writer’s cramp and musician’s dystonia). Task specificity refers to the occurrence of the dystonic movements or posturing within the context of a specific pattern(s) of movement [3]. BoNT injection is presently the most effective therapy for the management of focal task-specific dystonia. We include a review of six selected controlled studies demonstrating efficacy of BoNT.

## 2. Writer’s Cramp: History, Clinical Presentation and Epidemiology 

Writer’s cramp is a task-specific focal hand dystonia (FHD) affecting the upper extremity, occurring during the act of writing [4]. It is one of the most common forms of focal hand dystonia, with onset in the fourth decade of life [5]. It affects men with slightly higher prevalence than women, with an incidence of 2.7/1,000,000 per year [6]. Family history is reported in 5–20% of patients with writer’s cramp, with recent studies showing the possibility of genetic variants contributing to the development of various forms of focal hand dystonia, including writer’s cramp and musician’s dystonia [7]. 

Onset of symptoms is usually insidious, and slowly progresses over months. Symptoms of writer’s cramp begin when grasping a pen, and are characterized by an alteration of grip—usually noted to have the pen resting at the metacarpophalangeal joint (MCP) or anterior to it. Other times, the writer may exhibit hyperflexion of the distal interphalangeal joint with hyperextension of the proximal interphalangeal joints [4]. The wrist may also demonstrate hyperflexion, with patients reporting difficulty with pen/pencil grip, usually requiring additional force or effort to grip the utensil. Handwriting may be difficult to read, with the pen/pencil often jerking across the paper [8].

Writer’s cramp can be further classified into simple, complex and progressive. When present only with the task of writing, the dystonia is classified as simple. Once it extends to other manual tasks, including eating, typing, and shaving, it is then labeled progressive writer’s cramp. Complex writer’s cramp is noted at initial onset of dystonia with multiple manual tasks affected [4]. Approximately 25% of cases of writer’s cramp may go on to spread proximally, or begin to involve the unaffected limb, with rare spontaneous remissions (5% of the time) [9].

## 3. Musician’s Dystonia: History, Clinical Presentation and Epidemiology

Musician’s focal hand dystonia (MFHD) is a painless task-specific dystonia, presenting with involuntary movements, abnormal postures and loss of fine motor dexterity, which affects learned, technical bimanual movements, predominantly in the context of playing a musical instrument [10]. MFHD less commonly affects the amateur musician but is instead associated with prolonged practice, with typical age of onset in the fourth decade of life, usually at the peak of a musician’s career. For the affected individual, focal dystonia is very disabling, and may terminate musical careers. It affects men more than women, by a ratio of 4:1 [10]. Some studies indicate that MFHD is present in approximately 1–2% of professional musicians during their career, although prevalence rates are variable [11].

The dystonia is classified according to the task involved. Pianist’s or violinist’s cramp may affect the control of the finger, hand or arm movements, whereas embouchure dystonia involves coordination of the lips, tongue, facial and cervical musculature, as well as breathing while playing brass or wind instruments [12]. Typically, MFHD is painless, although muscle aching has been reported with prolonged spasm. Symptoms may come on as subtle loss of control with fast passages, episodic finger curling in percussionists, fingers sticking on the keys of a piano, involuntary flexion of thumbs with handling bows in string instrument players, or impaired control of embouchure in a woodwind player. Most musicians will attribute these subtle imperfections to poor practice, proceed to augment their efforts with more repetition, which will often exacerbate their problem [10]. 

The clinical evaluation for therapeutic intervention in these patients requires the inclusion of the instrument at the clinical encounter. Specific attention to the movements involved in the dystonic limb is crucial in muscle selection for BoNT injection. Often, video recordings of the musician performing should occur during the clinical encounter. The clinician, who may not be well versed in the art of the instrument, will need to rely on the musician to comment on where the key is not properly played. We discuss therapeutic intervention, with presentation of a narrative review of select, albeit limited, trial data for the use of BoNT in the management of focal task-specific dystonia.

## 4. Therapeutic Overview

Current therapeutic interventions for the management of focal task-specific dystonia include oral pharmacologic agents, chemodenervation with BoNT injection, surgery and physical therapy [13]. Oral pharmacologic intervention is often poorly tolerated due to systemic side effects, such as sedation, dry mouth, urinary or gastrointestinal adverse effects. Oral agents include anticholinergic drugs, such as trihexyphenidyl, anticonvulsants, such as primidone and phenytoin, as well as skeletal muscle relaxants, such as baclofen [14]. Surgical options, such as ventro-oral thalamotomy, have been performed in medical refractory cases [15]. 

At present, chemodenervation using BoNT-A has been the therapeutic intervention of choice in the management of focal task-specific dystonia (FTSD). It is administered, directly into muscle, which limits the possibility of systemic side effects. We will focus the rest of this review on the various included controlled studies demonstrating efficacy of the use of BoNT in FTSD, inclusive of muscle selection, techniques for toxin administration, as well as the safety/efficacy of this therapeutic intervention. 

Briefly, BoNT targets a specific SNARE protein for degradation at the neuromuscular junction, thus preventing cholinergic neurotransmitter release at the peripheral nerve terminal. Without the ability to release acetylcholine at the neuromuscular junction, the neuron is effectively silenced which results in muscle paralysis [16]. There is increasing evidence that BoNT may also act peripherally at gamma motor neurons, working to reduce the afferent sensory input from muscle spindles to the central nervous system, thus altering sensorimotor pathways [17]. Given the effect of BoNT with resulting paralysis of limb strength, an expert injector requires the proper restraint in use of toxin to reduce dystonic symptoms, while avoiding concomitant weakness in limb motor function [18]. 

## 5. Treatment Challenges and Pitfalls

The remainder of this review will focus on the use of BoNT for the management of FTSD. There have been several randomized, double-blind, controlled studies investigating the use of BoNT-A in the management of limb dystonia [19,20,21,22,23]. Conducting controlled studies in this patient population is difficult as both the patients, as well as outcome measures, are vastly variable. Reliably assessing the drug effect in an objective fashion is also challenging. The nature of the task specificity of each dystonia is also difficult to generalize more broadly. Outcome measures have included impact of quality of life and treatment response to injection, as measured by the patient’s interpretation of degree of dystonic movement [24]. Though literature exists on toxin selection for management of blepharospasm and cervical dystonia, we currently have no Class I studies comparing the various toxin formulations specifically for use in the management of FTSD [25]. Specifics regarding injection technique, muscle targeting, and toxin dosing remain areas that merit ongoing exploration.

## 6. Methods for Review of the Literature

Though limited numbers of trials have been performed, we conducted a review of the literature selected from the *MEDLINE* and ClinicalTrials.gov databases in accordance with Preferred Reporting Items for Systematic Reviews and Meta-Analyses (PRISMA) guidelines, with search period up to 25 July 2021. The search strategy included the following keywords: musician’s dystonia AND botulinum toxin, as well as writer’s cramp AND botulinum toxin. A single investigator searched the potentially eligible papers. All studies included examined the efficacy and safety of BoNT injection for the indications of writer’s cramp or musician’s dystonia, regardless of sample size. Studies using only the BoNT-A serotype were included. The selected studies were performed from 1991 to 2000, with the inclusion of 139 patients across the six selected studies, with 99 individuals affected by writer’s cramp, and 40 individuals with other task-specific focal dystonia, inclusive of musician’s dystonia. The age range included was 20–80 years old. We present specifics of each study below in Table 1.

## 7. Results: Trial Data

In 1992, Yoshimura et al. performed a placebo-controlled, blinded study to investigate the efficacy of BoNT-A in focal limb dystonia. Specifically, 17 individuals—ten with occupational cramps, three with idiopathic dystonia unrelated to task, and two with post-stroke and parkinsonian dystonia—were evaluated using graded doses of toxin injection. Muscle selection was performed using EMG at rest and then during task performance. Improvement occurred in 82% of patients following at least one dose of toxin, with effect lasting 1–4 months. Objectively, however, they were unable to demonstrate toxin efficacy as compared to placebo on dystonic movement during task [23]. 

The following year, Tsui et al. performed a placebo-controlled, blinded study on 20 individuals with writer’s cramp to determine the efficacy of BoNT-A. The primary end point was the measurement of pen control between the treatment vs. placebo group following two treatments, with assessments performed before treatment, at week 2 and week 6 following each treatment. The group demonstrated that 12 out of 20 individuals had improvement in pen control following BoNT-A injection, though only 4 of 20 had improvement in writing. BoNT-A appeared to most benefit individuals with dystonic movements involving the wrist joint [21]. 

In 1995, Cole et al. performed a double-blind, placebo-controlled study of BoNT-A effect on dystonic limb movements in individuals with focal dystonia (*n* = 10), including writer’s cramp (*n* = 6), sternographer’s cramp (*n* = 2) and musician’s dystonia (*n* = 2). Specifically, they assessed treatment response using three measures: 1. Subjective rating of intervention efficacy on dystonia. 2. Objective strength assessment (using Medical Research Council scale), as well as timing and rate of error in writing samples (for 6 individuals with writer’s cramp), rate of error (for 2 individuals with sternographer’s cramp), and performance rating by professional musicians (for 2 individuals with musician’s dystonia). 3. Blinded physician rating using video performance. The group found that 10 out of 10 individuals had greater subjective improvement with BoNT-A as compared to placebo [20]. 

Wissel et al. performed a blinded study evaluating local toxin injection in the efficacy of writing performance in 31 individuals with writer’s cramp. They utilized computer-assisted analysis scales to calculate writing speed, and objectively measured the baseline writer’s cramp rating scale scores (WCRS), in addition to writing speed. The mean toxin dose used was 133.2 units (U) injected into the forearm muscles (either flexors or extensors), using EMG guidance. They performed a total of 124 injections over one year, reporting 76% of the total number of injections produced an improvement from baseline WCRS. The most commonly reported side effect was weakness. WCRS was assessed using videotape by blinded reviewers (4 raters in total), showing good reliability between raters, with significant improvement in scores (*p* < 0.001). The speed of pen movements also showed significant increase following treatment, which correlated to patients’ subjective best-effect recordings (*p* < 0.05), with correlation in WCRS subscores, thus further confirming the validity of the scale used [22]. 

In 2007, Kruisdijk et al. performed a double-blind, randomized, placebo-controlled trial in 40 individuals with writer’s cramp, to determine the efficacy of BoNT-A injections. The primary end point was the patient’s answer to the question: “Considering all advantages and disadvantages of this treatment, is the improvement such that you wish to continue this treatment or not?” They also assessed additional secondary endpoints, including the visual analogue scale (VAS) for handwriting; symptom severity scale (SSS); functional status scale (FSS), writer’s cramp rating scale (WCRS), and writing speed. The study found that 70% of patients (14 of 20 individuals) receiving BoNT-A reported benefit and chose to continue treatment, as compared to 31.6% (6 of 19 individuals) in the placebo group. Using their secondary endpoints, they found clinical rating scales were significantly in favor of the BoNT-A group. Side effects reported included mild hand weakness, and local injection site pain [19].

Specific studies in musician’s dystonia are even more difficult to perform, given the variable nature of the presentations. In 2021, Frucht et al., presented data from a double-blind, placebo-controlled crossover study of inco-BoNT-A in focal musician’s hand dystonia. The group randomized 21 patients with focal musician’s dystonia to one of two cycles of treatment, with patients injected at day 1, followed by week 2 and 4 with booster doses (at the injector’s discretion). At week 8, dystonia severity and musical performance were rated as primary end points by blinded raters using video. Following this, the patients in cycle 1 were crossed to and subject’s intervention arm for cycle 2, which was performed at week 12 following initiation of cycle 1. The primary outcome measures for active drug (BoNT-A) at week 8 was shown to have improvement of both dystonia severity (*p* = 0.04) as well as musical performance (*p* = 0.027) as compared to the placebo arm. Raters were blinded and analysis performed using video. Minimal, non-disabling weakness was observed, using dynamometry. Additionally, serum neutralizing antibodies were collected at each visit. These data were presented at the virtual Movement Disorder Society meeting in 2021 [26]. 

## 8. Practical Approach for Treatment 

Given the complexity and highly individualized need for therapeutic intervention with BoNT-A injections for focal task-specific dystonia, a generalizable injection schema and specific guidelines are difficult to standardize. Pooled efficacy from the selected study does reveal response rate of 76% of all patients included across the indications of writer’s cramp, as well as the various focal task-specific dystonias, inclusive of musician’s dystonia. Dressler et al. presented consensus guidelines for toxin therapy, providing general algorithms and dosing tables for the indications of dystonia and spasticity. This group analyzed 11 years of data from 420 dystonia patients seen at the Movement Disorders center at the Hannover Medical School in Germany (50 individuals had writer’s cramp and 10 had arm dystonia). The group also looked at BoNT injection in 240 patients with spasticity. In total, 1831 BoNT injections spread over 36 target muscles were analyzed for dystonia. The group used only ona-BoNT-A and inco-BoNT-A formulations [27]. Much like in the aforementioned studies, this group identified target muscles based on the clinical examination, pathological positioning and movements during the exam. They delivered an average of 70.3 U of toxin to the involved target muscles (average 2.5 muscles), most often targeting the flexor digitorum superficialis (48%), flexor carpi ulnaris (42%), extensor carpi ulnaris (34%), extensor carpi radialis (30%), flexor digitorum profundus (30%), flexor pollicis longus (28%), flexor carpi radialis (12%), pronator teres (8%), extensor indicis (8%), and extensor pollicis muscles (6%). The group used ultrasonography or EMG for guidance, noting that dose ranges were highly individualized, ranging from 9.3 U in smaller muscles such as the extensor pollicis, to 35.4 U in larger muscles, such as the extensor carpi ulnaris muscle. In reviewing the select literature above, we propose the following practical approach for the injection of BoNT-A for the management of focal task-specific dystonia. 

### 8.1. Process of Muscle Selection in Toxin Administration for Task-Specific Dystonia

Given the specificity of movement in FTSD, it is critical to appropriately select the muscles of interest. The clinical examination is the first and perhaps, most critical evaluation, in helping to select the appropriate dystonic muscles. Attentive visual examination of the task, with repeated evaluation is necessary, often with assistance of video guidance. The musician must have the instrument and be evaluated by a skilled, carefully observant neurologist. Additional techniques can include surface electrode recording during performance of the task or surface electromyography (EMG) to evaluate for the rhythmic discharge of affected muscles. Most often, the musician or writer will report which movement is difficult to perform or release during the action. Once muscle selection is performed, the decision of toxin dose is also important and remains far less standardized. Dressler et al. do provide guidance on the ‘therapeutic window’ of different target muscles [27]. They note a narrow therapeutic window exists for smaller muscles, such as the finger extensors, peri-oral musculature, and finger flexors; a medium therapeutic window for the neck muscles, and a wider window for the orbicularis oculi musculature. They also comment on dose modification based on the toxin injection effect on the pathologic muscle activity as compared to its effect on muscle function/strength, as the ultimate objective of the expert injector is to ‘hit the right muscle with the right dose.’

### 8.2. Techniques for Botulinum Toxin Administration for Task-Specific Dystonia

Once appropriate muscle selection is performed, optimization of the injection technique is the priority. The studies noted in Table 1 utilized a combination of surface anatomy, surface electromyography and visualization of muscle with neuromuscular ultrasonography. Frucht et al. performed muscle localization using dual guidance, both with visualization of muscle with ultrasonography, and further focal localization with application of electrical stimulation to the muscle of interest for fascicular localization. For example, in a tabla player with focal dystonic flexor movement of the distal interphalangeal joint, BoNT-A injection was specifically administered to the fascicle of digit 2 of the flexor digitorum profundus muscle. This allows for the lowest dose of toxin administration into the target muscle, thus limiting excess weakness of the dystonic muscle. Additionally, this dual localization technique can further minimize the risk of toxin diffusion to, and potential weakness of, the adjoining muscles. 

### 8.3. Safety and Efficacy of Toxin Use in Task-Specific Dystonia

Side effects of BoNT-A are related to the potential of toxin to diffuse from injected muscles to nearby muscles or structures, which can result in the inadvertent weakness of muscles not meant for injection. Patients need to be educated on the risk of possible excess weakness, which is usually present within the first few weeks following injection. We have found that the optimal balance may be achieved with several weeks of non-disabling weakness of targeted muscles, followed by several months of dystonia relief. 

Relative contraindications to BoNT-A therapy include pregnancy, known history of impaired neuromuscular junction transmission (i.e., myasthenia gravis), myopathy and presence of prior paresis [28]. Most commonly experienced adverse effects include injection site reaction, and excess weakness which is dose dependent. Systemic side effects, such as dyspnea, is rare and requires > 10 times the dose typically used in practice. Patients may experience the formation of neutralizing antibodies, though this is reported in only 0.5–5% of patients treated with BoNT-A [29]. Specific targeting using dual localization as noted above can minimize the risk of side effects as this can limit dosing requirement, and target muscles with fascicular specificity. 

## 9. Conclusions and Future Directions

In summary, BoNT-A injections should be considered as an integral therapeutic option for the management of focal task-specific dystonia, especially in individuals who have demonstrated lack of benefit or adverse side effects with oral pharmacologic agents. After careful evaluation of the limb movements with task performance, inclusive of instrument performance, for musicians with dystonic movements, the expert clinician must use a specific localization and injection schema to target the muscles of interest. Targeting techniques continue to be areas that merit further research and sophistication, as more targeted muscle injection can help reduce toxin side effect and the over-weakening of neighboring muscles that are not involved in the dystonic movement during said task. Toxin dosing remains an area of active research and further work in exploring toxin dosing and efficacy/differences of efficacy of toxin formulations remains to be performed. 

## Figures and Tables

**Table 1:** Summary of Studies Using Botulinum Toxin-A in Focal Task-Specific Dystonia.

Study	Study Design	Study Objective	Sample Size	Study Method/Endpoints	Results
Frucht et al., 2021 [*NCT02107261*]	Double blind placebo controlled, crossover study of incobotulinumtoxin-A in focal musician’s hand dystonia	Determine safety/efficacy of incobotulinumtoxin-A	***n* = 21** (musician’s dystonia)	Cycle 1: Patients injected at day 1, followed by boosters at week 2 and 4 at injector’s discretion Primary efficacy at week 8 (measured by dystonia severity scale and musical performance) Cycle 2: Week 12, repeat injection with cross over design	-Primary outcome measure for active drug week 8 in comparison to baseline by blinded video rating yielded *p* = 0.04 for dystonia severity and *p* = 0.027 for musical performance -Minimal non-disabling clinical weakness by dynamometry was observed -serum neutralizing antibodies collected at each visit, results pending
Kruisdijk et al., 2005	Double-blind, randomized, placebo controlled trial	Determine efficacy of BoNT-A injections in patients with writer’s cramp	***n* = 40** (writer’s cramp)	Patients randomized to treatment with toxin v placebo over 2 sessions (over 12 weeks), with the following outcome measures: 1. Primary: patient’s answer to the following question: considering all advantages and disadvantages of this treatment, is the improvement such that you wish to continue this treatment or not? 2. Secondary: -visual analogue scale (VAS) for handwriting -symptom severity scale (SSS -functional status scale (FSS) -writer’s cramp rating scale (WCRS) -writing speed	-14 of 20 patients (70%) receiving BoNT-A reported a beneficial effect and chose to continue treatment, versus 6 of 19 patients (31.6%) in the placebo group (*p* = 0.03). -The changes on most of the clinical rating scales were significantly in favor of BoNT-A -Side effects: mild hand weakness, and pain at the injection site.
Wissel et al., 1996	Blinded study evaluating local toxin injection	Quantify treatment efficacy using writing performance and computer assisted analysis scales of writing speed	***n* = 31** (writer’s cramp)	-Assessed baseline WCRS (writer’s cramp rating scale score) and computer based writing speed analysis followed by repeat analysis at the time of patient’s subjective best response at follow-up visits (graded—0% no change to 100% uninterrupted writing) -Mean dose of aboBoNT-A was 133.2U between two forearm muscles	-Of all 124 injection sessions during mean follow up of one year, 76% produced a good improvement -The most common side effect was weakness (72% of the follow up visits) -The WCRS scores as assessed by a blinded videotape review by four independent raters showed good reliability between raters and a significant improvement after treatment (*p* < 0.001) -The speed of pen movements showed a significant (*p* < 0.05) increase after treatment at subjective best effect recordings and a significant correlation with WCRS subscores, documenting the validity of the scale
Cole et al., 1995	Double blind placebo controlled study of botulinumtoxin injection for focal dystonia (writer’s cramp, stenographer’s cramp and musician’s dystonia)	Determine clinical response to toxin v placebo in focal limb dystonia	***n* = 10** (6 writer’s cramp, 2 stenographer’s cramp, 2 musician’s dystonia)	Assessed response using 3 measures:1. Subjective rating by patient report on dystonia efficacy 2. Objective strength assessment (MRC), timing and rate of error in writing sample for writer’s cramp; rate of error in stenographer’s cramp, and performance rating by professional musicians for musician’s dystonia 3. Blinded physician rating using video performance	-10 patients had greater subjective improvement with botulinum toxin than with placebo -Two patients failed to have a better response to botulinum toxin than to placebo, with subjective reports further confirmed by objective testing
Tsui et al., 1993	Double-blind, placebo-controlled study	Determine efficacy of botulinumtoxin-A in patients with writer’s cramp	***n* = 20** (writer’s cramp)	-Measured pen control in patients following two treatments, three months apart (toxin v. normal saline injection) -Patients assessed before each treatment, at week 2 and week 6 following each treatment	12 of 20 patients had improvement in pen control following BoNT-A injection, but only 4 out of 20 had improvement in writing -those with wrist-joint deviation seemed to benefit the most
Yoshimura et al., 1992	Placebo-controlled, blinded study	Investigate efficacy of botulinumtoxin in focal limb dystonia	***n* = 17** (10 occupational cramps, 3 idiopathic dystonia unrelated to activity, 2 with post-stroke and parkinsonian dystonia)	Measure subjective improvement following injection in placebo v. control group. Muscle selection performed via EMG of muscles during task -3 injections of graded doses of toxin (5–10 U, 10–20 U and 20–40 U per muscle)	-Report that subjective improvement noted after 53% of injections, noted to be substantially improved in 24% of patients. -One patient improved following placebo. -At least 82% of patients demonstrated subjective improvement following one dose, with effect lasting 1–4 months -Objectively, however, study failed to demonstrate significant improvement in toxin v placebo group -adverse side effect was weakness (53%)

## Data Availability

The data presented in this study are openly available in *PubMed* at: DOI: 10.1136/jnnp.2005.083170, reference number [19]; DOI: 10.1002/mds.870100411, reference number [20]; DOI: 10.1212/wnl.43.1_part_1.183, reference number [21]; DOI: 10.1136/jnnp.61.2.172, reference number [22]; DOI: 10.1212/wnl.42.3.627, reference number [23].
